# Effects of Modifying Supportive Care Medications in Combination Therapy with Pertuzumab, Trastuzumab, and Taxane Anticancer Drugs

**DOI:** 10.3390/pharmacy13060168

**Published:** 2025-11-17

**Authors:** Mina Takagi, Shinichiro Maeda, Makiko Maeda, Yasushi Fujio, Sachiko Hirobe

**Affiliations:** 1 Laboratory of Clinical Pharmacology and Therapeutics, Graduate School of Pharmaceutical Sciences, The University of Osaka, 1-6 Yamadaoka, Suita 565-0871, Osaka, Japan; 2 Department of Pharmacy, The University of Osaka Hospital, 2-15 Yamadaoka, Suita 565-0871, Osaka, Japan; 3 Laboratory of Molecular Pharmaceutical Science, Graduate School of Medicine, The University of Osaka, 2-2 Yamadaoka, Suita 565-0871, Osaka, Japan; 4 Laboratory of Clinical Science and Biomedicine, Graduate School of Pharmaceutical Sciences, The University of Osaka, 1-6 Yamadaoka, Suita 565-0871, Osaka, Japan; 5 Integrated Frontier Research for Medical Science Division, Institute for Open and Transdisciplinary Research Initiatives, The University of Osaka, 1-1 Yamdaoka, Suita 565-0871, Osaka, Japan

**Keywords:** trastuzumab, pertuzumab, taxane, infusion-related reaction, premedication

## Abstract

Chemotherapy for breast cancer includes pertuzumab and trastuzumab regimens with docetaxel (PHD) or paclitaxel (PHP). Current approaches for using supportive care drugs to manage the side effects of PHD and PHP are unclear. Here, we investigated the occurrence of side effects before and after supportive care medications were modified by discontinuing antipyretic analgesics. We retrospectively analyzed adverse events that occurred within 24 h of treating 76 patients with PHD or PHP. The frequencies of adverse effects in the groups before and after modification did not differ significantly (45.5% [15/33] and 44.2% [19/43], respectively). Severity also did not significantly differ between the groups. Therefore, discontinuing antipyretic analgesics as supportive care drugs had little effect on the frequency of side effects. Symptoms of feeling hot, pyrexic, and flushed were frequent, and their severity increased in the group after the support drugs were modified. Discontinuation of supportive care medications, including antipyretic analgesics, might affect the severity of certain symptoms and lead to the development of side effects that require medical intervention. Overall, our findings indicate the need to consider premedication with antipyretic analgesics, including further analysis of the risk factors that can predict symptoms.

## 1. Introduction

Chemotherapy for human epidermal growth factor receptor type2 (HER2)-positive breast cancer involves pertuzumab (PER) and trastuzumab (TRA) with docetaxel (DTX) (PHD) or paclitaxel (PTX) (PHP) regimens. The humanized monoclonal antibodies PER and TRA target the HER2 protein and exert antitumor activity against HER2-positive cells. Infusion-related reactions (IRRs) are adverse events similar to hypersensitivity or allergic reactions that occur most frequently during or up to 24 h after antibody drugs are infused. The proposed mechanism of IRRs is that monoclonal antibodies interact with target blood and tumor cells, which induces cytokine release. These cytokines then diffuse through the systemic circulation, resulting in various characteristic symptoms, such as fever, chills, rash, flushing, and pruritus [1]. The frequency of IRR with TRA is 40% after the first dose, and this decreases after a second dose [2]. Although IRR is often mild, it occasionally causes serious lung injuries, such as respiratory failure, which requires appropriate management [3]. However, the value of supportive care agents for preventing TRA-induced IRR remains unexplored, and clear recommendations for supportive care agents are not listed in the guidelines of the European Society of Clinical Oncology or in package inserts in Japan [4].

The anticancer taxane drugs DTX and PTX inhibit tumor cell growth by blocking microtubule depolymerization and are used to treat several types of cancers. The most common effects of DTX and PTX are allergic symptoms. Among others, anaphylaxis often occurs within 10 min of infusing a first or second dose [4]. According to the package insert, the frequency of allergies to DTX is <5–50%, and although most symptoms are mild, they can become anaphylactic during infusions and require attention [5]. Similarly to DTX, PTX causes respiratory symptoms and skin involvement, usually within 15 min after initiating infusions [6]. Generally, PTX carries a higher risk of allergic reactions than DTX [7]. Corticosteroids are recommended as supportive care when DTX is injected, whereas patients must be premedicated with corticosteroids and antihistamines before PTX administration.

The occurrence of IRRs is significantly suppressed when dexamethasone is administered before TRA [8]. However, the regimens in that study varied, and some patients were treated with taxanes in addition to TRA. Supportive care drugs during PHD therapy can effectively suppress DTX-induced allergies when administered before PER and TRA infusion [9,10]. Thus, supportive care medications are not standardized, and their association with IRR and allergy development in PHD and PHP therapies remains unclear.

We investigated supportive care medications for PHD and PHP regimens, the occurrence of IRR, and allergic symptoms at The University of Osaka Hospital between August 2018 and November 2023. In April 2021, supportive care medications were modified to reduce IRR and allergic symptoms based on the recommendations in the package inserts and other documents. We compared the effects of supportive care medications on early-onset IRR and allergic symptoms before and after modification.

## 2. Materials and Methods

### 2.1. Patients and Regimens

**Figure 1:**
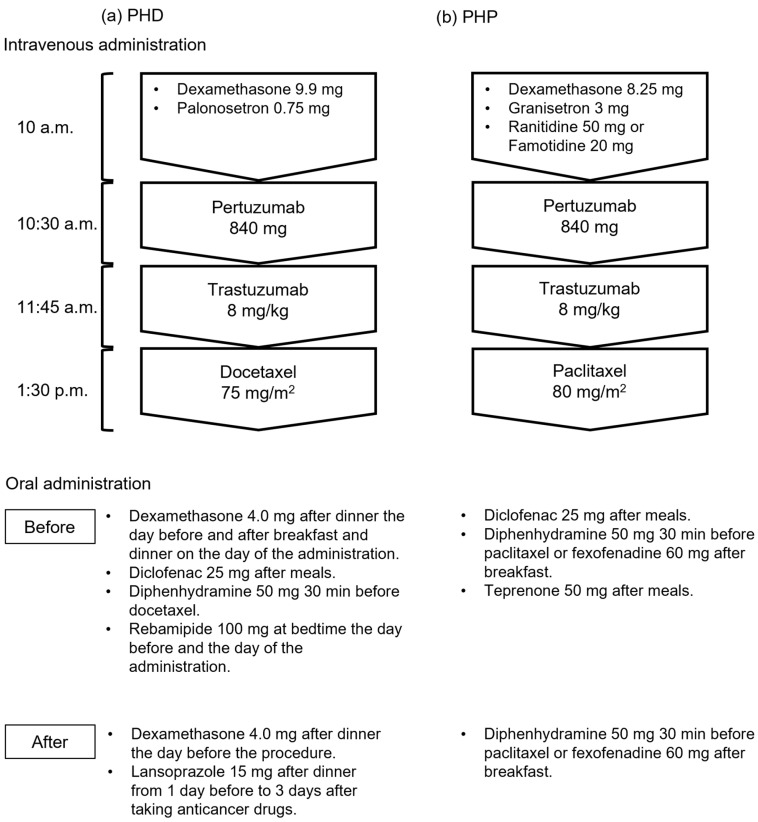
Regimens before and after supportive care drugs were modified. (**a**) Therapy with pertuzumab, trastuzumab, and docetaxel (PHD); (**b**) therapy with pertuzumab, trastuzumab, and paclitaxel (PHP).

We investigated the medical records of inpatients with breast cancer who received their first dose of PHD or PHP therapy between August 2018 and November 2023 at The University of Osaka Hospital. We excluded patients with other types of cancer and those with incomplete data. Seventy-six patients were treated in this study, of whom 33 and 43 were in the groups that were treated before and after supportive care medications were modified, respectively. Figure 1 shows intravenous and oral administration of PHD and PHP regimens. Some doses of dexamethasone, diclofenac, and diphenhydramine were discontinued after the modifications, and the gastric medication was switched from rebamipide to lansoprazole in the PHD regimen. Diclofenac and concomitant teprenone were also discontinued in the PHP regimen.

### 2.2. Collection of Medical Information

Patient background information about age, sex, weight, body mass index (BMI), cancer subtype, TNM classification, history of surgery, anthracycline chemotherapy, allergies, regimens, supportive care medications, stage, hormone receptor expression, and side effects was collected from medical records. Except for constipation, which could not be ruled out as causally associated with PER, TRA, DTX, or PTX treatment, we defined side effects as adverse events that occurred within 24 h of PER administration. Symptoms were evaluated based on information from medical records and indicated as the preferred terms using MedDRA/J Version 27.0; the severity of side effects was classified according to the IRRs in CTCAE v. 5.0.

### 2.3. Statistical Analysis

We compared the two groups before and after modifying supportive care drugs using JMP^®^Pro v. 17.1.0 (JMP Statistical Discovery LLC., Cary, NC, USA). Continuous functions were analyzed using Wilcoxon rank sum tests, and binary variables were assessed using Pearson’s chi-square and Fisher’s exact tests. All values with *p* = 0.05 were considered statistically significant.

## 3. Results

### 3.1. Characteristics of Patients

Of the 76 patients, 33 and 43 were in the group treated before and after modification, respectively. Table 1 shows the characteristics of the patients. Cancer stage, hormone receptors, and BMI did not significantly differ between the groups before and after modification. However, significantly more patients were older, treated with PHD, and were in postoperative conditions in the group treated after modification.

### 3.2. Frequency of Side Effects Before and After Modification Supportive Care Medications

**Figure 2:**
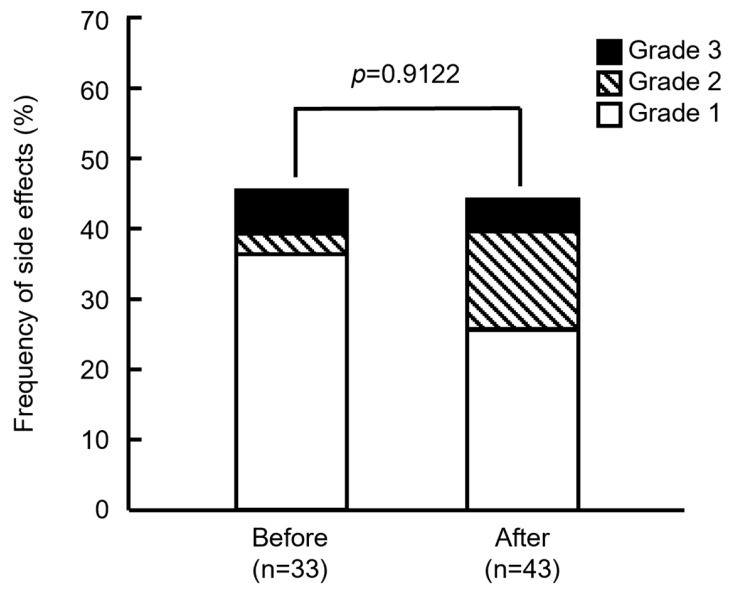
Frequency of side effects in patients before and after supportive care drugs were modified. *p* = 0.9122 (Pearson’s χ^2^ test). Grade indicates the highest grade of symptoms exhibited by each patient.

Figure 2 shows that the frequencies of side effects did not significantly differ between the groups before and after modification (45.5% [15/33] and 44.2% [19/43]; *p* = 0.9122, Pearson’s χ^2^ test). Severity also did not significantly differ between the groups (*p* = 0.3310, Fisher’s exact test). Based on the assessment of IRRs, severity was determined as Grade 2 if the anticancer drugs were tapered or suspended, or reactions were medicated, and as Grade 3 when symptoms persisted, or medications were repeated. Among the patients in the groups treated before modifications, 2 showed Grade 3 (6.06%), 1 showed Grade 2 (3.03%), and 12 showed Grade 1 (36.4%) side effects. In the group treated after modifications, 2 showed Grade 3 (4.65%), 6 showed Grade 2 (14.0%), and 11 showed Grade 1 (25.6%) side effects. These results showed that the number of Grades 2 or 3 side effects increased in the group treated after the drugs were modified. One patient with Grade 2 or 3 in the group after the support drugs were modified could not resume treatment because of continued abdominal pain and headache, but she started PHD therapy one month later. Others resumed treatment and were completed by the end of the day.

### 3.3. Symptoms

**Figure 3:**
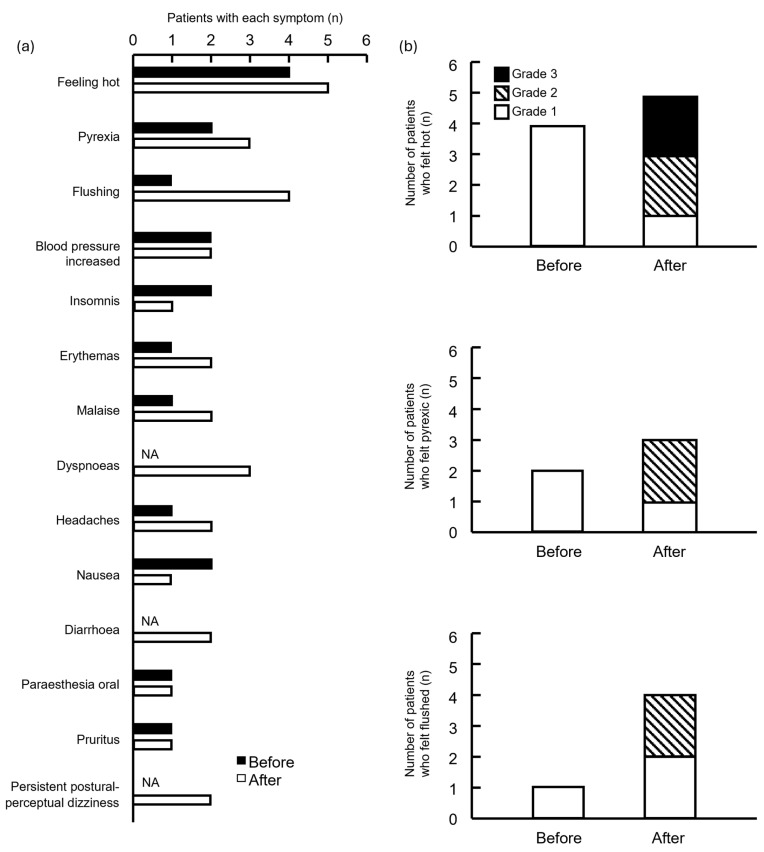
Number of symptomatic side effects in patients treated before and after supportive care drugs were modified. (**a**) Patients and symptoms; (**b**) number of patients who felt hot, pyrexic, and flushed. NA, not applicable. Grade indicates the highest grade of symptoms exhibited by each patient.

The frequency of feeling hot, pyrexic, and flushed was high in both groups (Figure 3a). Although the severity of these symptoms increased in the group after drug modification, the difference was not significant (Figure 3b).

### 3.4. Timing of Side Effects

**Figure 4:**
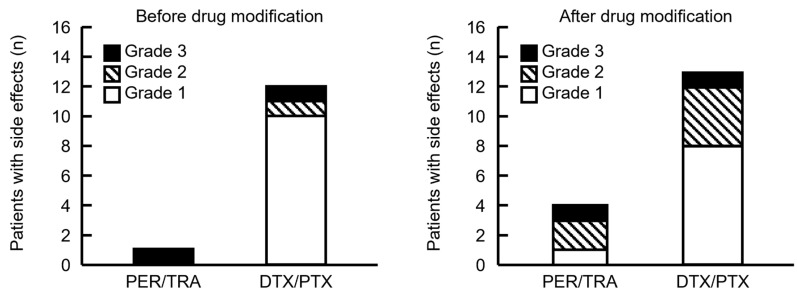
Comparison of the timing of side effects in patients before and after the supportive care drugs were modified. PER/TRA indicates the number of side effects that occurred before the administration of DTX or PTX. DTX/PTX indicates the number of side effects that developed after DTX or PTX administration. Four patients experienced side effects after DTX or PTX administration; however, the timing of onset was unknown and therefore not included in the figure. Grade indicates the highest grade of symptoms exhibited by each patient.

Antibody drugs that elicited IRR and taxane drugs associated with allergic symptoms were consecutively administered during PHD and PHP therapy. Therefore, we determined the drugs that caused side effects based on timing. Five and 25 patients developed side effects from the start of PER until the end of HER, and after the start of DTX or PTX administration, respectively. Therefore, many patients developed symptoms after taxane infusion. The frequency of side effects did not significantly differ before and after supportive care drugs were modified, and most of them occurred after starting taxane infusions (Figure 4). Although our cohort was small, the severity of side effects during PER/TRA was higher before and after the supportive care drugs were modified. However, more patients tended to develop side effects after modification.

Side effects observed after the administration of taxane drugs were also analyzed according to the treatment regimen to evaluate symptoms suggestive of hypersensitivity reactions to either DTX or PTX. Side effects following the initiation of DTX were observed in 3 of 5 patients (60%) and in 11 of 39 patients (28.2%) before and after supportive care drugs were modified. Side effects following the initiation of PTX were observed in 9 of 28 patients (32.1%) and in 2 of 4 patients (50%) before and after supportive care drugs were modified. Table A1 presents all side effects noted in each patient.

## 4. Discussion

### 4.1. Interpretation of Findings

A comparison of the characteristics revealed that more patients received PHD therapy and were older in the group after supportive care medications were modified. This might be attributed to changes in recommended regimens, such as revised breast cancer treatment guidelines, under which younger patients are now being treated with drugs other than PHD/PHP.

We found that the frequency of side effects and their severity did not significantly differ before and after drug modification. These results suggest that safety can be guaranteed even if some supportive care drugs are discontinued. The frequency of IRR might have been higher than previous findings because we included all events other than constipation, which could not be ruled out as being causally related to the administration of PER, TRA, DTX, and PTX [8,9,10,11,12,13,14,15,16].

Patients frequently felt hot, pyrexic, and flushed, and the severity of these symptoms increased in the group treated after drug modification. This suggested that partial discontinuation of supportive care medications, including the antipyretic analgesic diclofenac, might affect the severity of some symptoms and lead to the development of side effects requiring medical intervention. Future investigation should thus consider the need for premedication with antipyretic analgesics by analyzing risk factors that can predict the development of symptoms. Antipyretic analgesics were administered after every meal before we modified the supportive care medications. As some patients developed side effects after dinner, further studies are needed to determine the need to take antipyretic analgesics after every meal before starting taxane drugs.

We analyzed the drugs that caused side effects based on timing and found that more patients developed side effects after starting taxane therapy than antibodies. This indicated that the taxane drugs might have caused many of the symptoms. A similar number of patients in both groups developed adverse effects after initiating taxane infusion.

Although modification of supportive care drugs may have had minimal effects on the frequency of side effects of taxanes, evaluation was challenging because of the different proportions of patients in the two groups who received PHP and PHD therapy. Clinical applications have shown that allergic reactions to both PTX and DTX most commonly occur during the first administration. Although it remains unclear whether these reactions are caused by the taxane itself or by its formulation vehicle, they are typically considered non–IgE-mediated, with Cremophor EL and polysorbate 80 being the main causes in PTX and DTX, respectively [6,17]. Allergic symptoms, including skin symptoms, hypotension, and dyspnea, are similar for both agents [17,18]. Notably, PTX may pose a higher risk of allergic reactions than DTX; therefore, premedication with a greater number and dose is recommended for PTX [7,17]. We found that therapy with PHD was significantly more prevalent after the drugs were modified, and its effects must be considered. However, differences in regimen distribution between groups prevented statistical comparison of side effects within each regimen before and after the modification. In the PHD regimen, although many supportive care drugs administered before DTX infusion were discontinued, the frequency of side effects following DTX administration decreased, suggesting that supportive care drugs had minimal influence on DTX-related side effects. In the PHP regimen, the only modification was the discontinuation of diclofenac. Although the frequency of side effects following PTX administration appeared unchanged, their severity tended to increase, implying that diclofenac may help mitigate PTX-related side effects, which there are few reports suggest. However, owing to the limited sample size, further validation is needed to confirm this effect. Overall, the unchanged frequency of side effects after the modification suggests that treatment safety was maintained. Moreover, when analyzing data from both before and after drug modification, the frequency, severity, and symptoms following taxane administration were comparable between the PHD and PHP regimens.

Our small cohort developed severe side effects during antibody administration before and after drug modifications. However, a greater number of patients treated after drug modification developed severe side effects. Therefore, preventive measures against IRR with antibody drugs are important. In this study, the major modification in supportive care medications was the discontinuation of diclofenac. Nonsteroidal anti-inflammatory drugs (NSAIDs), including diclofenac, have potential roles in the prevention and management of IRRs. NSAIDs inhibit the activity of cyclooxygenase (COX)-1 and COX-2, thereby suppressing the synthesis of prostaglandins (PGs) [19]. Inhibition of PGE_2_ reduces NF-κB activity and decreases the production of cytokines such as interleukin-6 (IL-6) and tumor necrosis factor-α (TNF-α). In a murine model of SARS-CoV-2 characterized by cytokine upregulation, NSAID treatment reduced the production of IL-6 and TNF-α [20]. Although the mechanisms of adverse events caused by monoclonal antibody therapies are diverse [21], IRRs are primarily believed to result from cytokine release triggered by interactions between monoclonal antibodies and their target molecules or various immune cells [1]. Patients showed IRRs accompanied by increased levels of cytokines such as IL-6 and TNF-α [22]. Therefore, NSAIDs can be used for IRRs involving cytokine amplification. NSAIDs alleviated adverse events associated with antibody therapies or reduced the frequency of IRRs [13,23]. Although large-scale randomized controlled trials demonstrating the efficacy of NSAIDs in reducing IRRs are lacking, their use as part of premedication regimens—alongside corticosteroids and antihistamines—appears to be reasonable. The corticosteroids dexamethasone and hydrocortisone may also help to prevent TRA-induced IRRs [8,14,16]. Overall, the present findings may facilitate appropriate drug selection to help prevent side effects. Regarding the appropriate timing of supportive care drugs, those used to prevent taxane drug allergies could be administered between antibodies and taxane drugs [9,10]; however, for IRR prophylaxis, they should be applied before infusing antibody drugs.

TRA’s patent has now expired and biosimilars have become available in Japan since August 2018. Although, results proved equivalence between the biosimilars and the reference product (Herceptin^®^; Genentech Co., Ltd., San Francisco, CA, USA) in efficacy and safety [24,25], they are not entirely identical because biologics are produced using animal cells, post-translational modifications result in heterogeneity. Given that real-world safety data remain limited, and all TRA used in this study was the reference product, comparative safety investigation between formulations is necessary.

A fixed-dose subcutaneous combination of PER and TRA (Phesgo^®^; Genentech Co., Ltd., San Francisco, CA, USA) has also become available in Japan to treat HER2-positive breast cancer since September 2023. The frequency of IRR with Phesgo^®^ was 4.0% [26] which was lower than that with the intravenously injected PER and TRA; however, subcutaneous injection is considered difficult to use in patients with low body weight or edematous thighs. Therefore, intravenous injections are likely to persist, and further investigation into IRR prophylaxis is necessary.

### 4.2. Limitations

The most significant limitation of this study was the presence of significant differences in baseline characteristics between the before and after modification groups (Table 1), which necessitated comparison after adjusting for confounding factors. Multivariate analysis should have been performed after baseline adjustment to strengthen the causal inference regarding the effect of the supportive care medication modification. However, this was not feasible owing to the small sample size. The sample size was not calculated in advance for this study. As a result of the retrospective investigation, only 76 patients with breast cancer receiving PHD or PHP therapy were enrolled. Although studies with larger sample sizes should be implemented, it is not feasible to increase the number of patients who received the PHD regimen before the modification at our hospital. Furthermore, because no other facilities have been identified that administer PHD or PHP therapy with the same supportive care drug protocols as ours, conducting additional analyses with an expanded sample size would be difficult.

Additionally, the supportive care medications used in the PHD and PHP regimens differed. Nevertheless, subgroup analyses could not be performed because of the uneven distribution of patients between groups. Therefore, the conclusion that safety was maintained “despite differences in the portions of regimens” is speculative without directly testing for an interaction between the regimen type and modification’s effect. Regarding the analysis of the drugs that caused side effects based on timing, most events occurred after the administration of the taxane drugs. Although this finding suggests that the taxane may have been the primary cause, delayed reactions caused by the antibody drugs may have occurred among the events observed after taxane administration. Therefore, the timing of onset alone is insufficient to definitively identify the drug responsible for the adverse event.

Other limitations include the retrospective design with inherent bias, and that it was conducted at a single hospital. No objective criteria are available to evaluate side effects, and subjective judgments by institutions and healthcare professionals may have caused variations. In particular, the evaluation of feeling hot is subjective. Furthermore, for four patients, it was unclear whether side effects occurred from the start of PER until the end of HER, or after the start of DTX or PTX administration. Therefore, further studies with standardized assessment criteria are needed. It is also possible that the supportive care medications may have caused some of the symptoms in our patients.

Moreover, these patients received adequate supportive care medications compared with those in other hospitals; therefore, the discontinuation of some supportive care medications probably did not affect their safety. Finally, this study only collected data on adverse events occurring within 24 h after anticancer drug administration. Given that a severe delayed reaction associated with TRA has been reported [27], and that taxane drugs induce allergic reactions even upon the second administration [6,28], it is essential to collect med- to long-term safety data.

### 4.3. Future Directions

We plan to determine initial responses, such as modifications in supportive care medications for specific IRR symptoms, to establish measures for preventing side effects of PHD and PHP therapy. We also plan to collect and analyze mid- to long-term safety data for PHD and PHP therapies in future investigations.

## 5. Conclusions

Within the limitations of this small-sample retrospective study with potential confounding factors, simplification of the premedication regimen did not lead to a significant increase in the overall frequency of adverse events. These findings need to be validated in larger, controlled studies prospectively.

## Figures and Tables

**Table 1:** Characteristics of patients treated before and after supportive care drugs were modified.

Patients		Before (*n* = 33)	After (*n* = 43)	*p*
Female		33 (100)	43 (100)	
Age (years)		49.5 (32.3–65.7)	59.8 (40.1–79.2)	<0.0001 *^,a^
Body Weight		56.0 (40.8–79.6)	55.8 (42.4–84.5)	0.8217 ^a^
Body Mass Index		22.0 (16.8–32.9)	23.1 (17.2–34.1)	0.7060 ^a^
Stage	I–III	29 (87.9)	41 (95.3)	0.3940 ^c^
	IV	4 (12.1)	2 (4.65)	
Purpose of chemotherapy (Stage I–III)	Neoadjuvant	23 (69.7)	23 (53.5)	0.0439 *^,b^
	Adjuvant	6 (18.2)	18 (41.9)	
History of anthracycline therapy	Yes	6 (18.2)	16 (37.2)	0.0698 ^b^
ER status	Positive	18 (54.5)	23 (53.5)	0.9270 ^b^
	Negative	15 (45.5)	20 (46.5)	
PgR status	Positive	12 (36.4)	20 (46.5)	0.3745 ^b^
	Negative	21 (63.6)	23 (53.5)	
Allergy	Yes	20 (60.6)	26 (60.5)	0.9901 ^b^
	No	13 (39.4)	17 (39.5)	
Regimen	PHD	5 (15.2)	39 (90.7)	<0.0001 *^,b^
	PHP	28 (84.8)	4 (9.3)	

Results are shown as median (range) or *n* (%). * *p* < 0.05 (^a^ Wilcoxon’s rank sum test; ^b^ Pearson’s chi-square test; ^c^ Fisher’s exact test). ER, estrogen receptor; PgR, progesterone receptor; PHD, pertuzumab, trastuzumab, and docetaxel; PHP, pertuzumab, trastuzumab, and paclitaxel.

## Data Availability

The data presented in this study are available on request from the corresponding author due to privacy or ethical restrictions.

## Abbreviations

The following abbreviations are used in this manuscript:

PHD	Pertuzumab, Trastuzumab, and Docetaxel
PHP	Pertuzumab, Trastuzumab, and Paclitaxel
HER2	Human epidermal growth factor receptor type 2
PER	Pertuzumab
TRA	Trastuzumab
DTX	Docetaxel
PTX	Paclitaxel
IRR	Infusion-related reaction
BMI	Body mass index

## Appendix A. Description of Side Effects for Each Patient

**Table A1:** Description of side effects for each patient.

Subject ID	Group	Regimen	Timing	Symptoms	Grade
1	Before	PHP	Unknown	Oedema	1
2	Before	PHP	Unknown	Blood pressure increased; Somnolence; Headaches; Nausea	1
3	Before	PHP	DTX/PTX	Blood pressure increased	2
4	Before	PHD	DTX/PTX	Malaise	1
5	Before	PHD	DTX/PTX	Initial insomnia; Pruritus; Erythema; Paraesthesia oral	3
6	Before	PHP	PER/TRA	Somnolence; Feeling abnormal; Tension; Discomfort	3
7	Before	PHP	DTX/PTX	Initial insomnia	1
8	Before	PHP	DTX/PTX	Palpitations; Feeling hot	1
9	Before	PHP	DTX/PTX	Feeling hot	1
10	Before	PHP	DTX/PTX	Pyrexia; Oropharyngeal discomfort	1
11	Before	PHP	DTX/PTX	Hyperhidrosis; Feeling hot	1
12	Before	PHP	DTX/PTX	Flushing	1
13	Before	PHP	DTX/PTX	Nausea	1
14	Before	PHP	DTX/PTX	Feeling hot	1
15	Before	PHD	DTX/PTX	Pyrexia	1
16	After	PHD	PER/TRA	Flushing	1
17	After	PHD	PER/TRA	Abdominal pain; Headaches	2
18	After	PHD	DTX/PTX	Initial insomnia	1
19	After	PHD	DTX/PTX	Erythema; Diarrhoea	2
20	After	PHD	DTX/PTX	Pyrexia	1
21	After	PHD	PER/TRA	Flushing; Paraesthesia oral; Feeling hot; Erythemas; Pruritus	2
22	After	PHD	DTX/PTX	Scintillating scotoma; Dyspnoea; Flushing	2
23	After	PHD	DTX/PTX	Malaise	1
24	After	PHD	DTX/PTX	Diarrhoea	1
25	After	PHD	DTX/PTX	Persistent postural-perceptual dizziness	1
26	After	PHD	DTX/PTX	Nausea	2
27	After	PHD	Unknown	Feeling hot	1
28	After	PHD	DTX/PTX	Periorbital swelling; Dyspnoea	1
29	After	PHD	DTX/PTX	Flushing	1
30	After	PHD	PER/TRA	Pyrexia; Feeling cold; Feeling hot	3
31	After	PHP	DTX/PTX	Feeling hot	2
32	After	PHD	DTX/PTX	Blood pressure increased	1
33	After	PHD	Unknown	Blood pressure increased; Headaches	1
34	After	PHP	DTX/PTX	Oxygen saturation decreased; Feeling hot; Sinus tachycardia; Pyrexia; Persistent postural-perceptual dizziness; Disorientation; Malaise	3

Before: the group treated before modifications. After: the group treated after modifications. Grade indicates the highest grade of symptoms exhibited by each patient.
